# Antibiotic consumption in children prior to diagnosis of asthma

**DOI:** 10.1186/1471-2466-11-32

**Published:** 2011-05-31

**Authors:** Fawziah Marra, Carlo A Marra, Kathryn Richardson, Larry D Lynd, Mark J FitzGerald

**Affiliations:** 1 Professor, Faculty of Pharmaceutical Sciences, University of British Columbia; Director, Pharmacy and Vaccine Services, British Columbia Centre for Disease Control, Vancouver, B.C., Canada; 2 Associate Professor and Director, Collaboration for Outcomes Research and Evaluation, Faculty of Pharmaceutical Sciences, University of British Columbia; Scientist, Centre for Health Evaluation and Outcome Sciences, Providence Health Research Institute, Vancouver, B.C. Canada; 3 Medical Statistician, Department of Public Health and Primary Care, University of Cambridge, Cambridge, UK; 4 Assistant Professor and Associate Director, Collaboration for Outcomes Research and Evaluation, Faculty of Pharmaceutical Sciences, University of British Columbia; Scientist, Centre for Health Evaluation and Outcome Sciences, Providence Health Research Institute, Vancouver, B.C. Canada; 5 Professor and Head, Division of Respiratory Medicine, Centre for Lung Health, Faculty of Medicine, University of British Columbia; Scientist, Centre for Clinical Epidemiology and Evaluation Vancouver Coastal Health Research Institute, Vancouver, B.C., Canada

## Abstract

**Background:**

Asthma is difficult to diagnose in children and at times misdiagnosis of an infection can occur. However, little is known about the magnitude and patterns of antibiotic consumption in children with asthma relative to those without asthma.

**Methods:**

Using population-based data, 128,872 children were identified with at least 6 years of follow-up. The adjusted rate-ratio (RR) of antibiotics dispensed to asthmatic as compared to non-asthmatic children was determined.

**Results:**

At age six, the RR of antibiotic consumption for asthmatics compared to non-asthmatics varied between, 1.66 to 2.32, depending on the year of asthma diagnosis. Of the 18,864 children with asthma at ages 2-8, 52% (n = 9,841) had antibiotics dispensed in the 6 months prior to their index date of asthma diagnosis. The RR of antibiotic consumption in the 1 month prior to asthma diagnosis compared to 5 months prior was 1.66 (95% CI 1.60-1.71). The RR was lower in males compared to females (1.58 vs 1.77), and lower in those who received antibiotics in the first year of life relative to those that did not (1.60 vs. 1.76).

**Conclusions:**

There is higher antibiotic consumption in children with asthma compared to those without asthma. The pattern of antibiotic use suggests that diagnosis guidelines are difficult to follow in young children leading to misdiagnosis and over treatment with antibiotics.

## Background

Studies performed in varying populations over the last 30 years have indicated that the prevalence of asthma has significantly increased [1,2]. Asthma rates are particularly high in children with one in eight school children being affected [3]. Asthma is difficult to diagnose - particularly in children as frequently their only symptom is coughing which may be mistaken for an upper respiratory tract infection in addition to the fact that they cannot participate in many of the standard diagnostic tests. The North American guidelines [4,5] for pediatric asthma management outline a stepwise approach to asthma management with the aim being to initiate control of asthma with regular anti-inflammatory therapy and monitor the level of control by use of rescue medication as well as symptoms, and in older children with assessment of airflow obstruction [6,7]. As such, patients should start treatment that is consistent with the initial severity of their asthma and then step down when control has been achieved. The availability of guidelines does not necessarily mean a high uptake by clinicians. Several studies have evaluated the use of asthma medications in adults and children and have shown that 40 to 60% of prescribing does not reflect the recommendations in guidelines [8-10].

In contrast to the use of asthma medications, antibiotics are over utilized in children and often prescribed for viral infections [11-15]. Our recent studies have evaluated the use of antibiotics in Canadian children and we showed that although overall antibiotic use had declined over a decade, their use in a Canadian population exceeds that of European children [11]. In addition, the use of antibiotics in children under 4 years of age, was double that of children between 10-14 years of age - and was primarily associated with upper respiratory tract infections, acute otitis media and bronchitis [12].

We know that antibiotics are not routinely used in the treatment of asthma - the asthma treatment guidelines clearly state that although symptoms associated with poor asthma control often overlap with symptoms associated with respiratory tract infections, clinicians should take care in differentiating between the two conditions. We conducted this study to determine the patterns of antibiotic consumption in children with asthma. Specifically, we wanted to determine if antibiotic consumption was greater in children who developed asthma compared to those who did not and to examine the rate of antibiotic consumption prior to the diagnosis of asthma.

## Methods

### Study Population

The longitudinal study cohort consisted of all live births in British Columbia (BC), Canada, from January 1997 through December 2003. Follow-up information was available from all data files up to December 2005.

### Data Sources

We used population-based, linked administrative health data that contained information on all physician services (known as MSP), hospital admissions, vital status information, socioeconomic status (SES) (determined through postal code and census classifications such as median neighbourhood income and split by quintiles therefore those in neighborhoods reporting the greatest 20% of incomes in the population will be in the 5th quintile) and outpatient dispensing episodes of prescription drugs (PharmaNet) to assess potential antibiotic exposures, potential confounders, and outcomes. Linkages among the various data files were achieved through the use of a personal health number unique to every BC resident; all traceable personal identifiers were removed to protect patient confidentiality. Ethics approval was obtained from the University of British Columbia Behavioral Research Ethics Board.

Information on dispensed asthma drugs and antibiotics between 1997 and 2005 was extracted based on the 2009 Anatomical Therapeutic Chemical Classification System (ATC) codes [16]. Asthma medications (ATC codes R03A-D) consisted of short-acting β-agonists, inhaled corticosteroids, ipratropium, or leukotriene receptor antagonists and ketotifen (ATC code R06AX17). Antibiotics (ATC code J01) were classified as penicillins, cephalosporins, macrolides, sulfonamide, or other classes.

Physician visits in the database were classified according to the 3-digit diagnostic code (International Classification of Diseases, Ninth Revision (ICD-9)) associated with that visit and the type of physician (e.g. general practitioner versus specialist). These visits were also extracted in the year prior to asthma diagnosis. In addition, any visits to an allergist, respirologist, or immunologist visit were obtained. Similar codes were used to classify hospitalizations.

### Asthma Definition and Index Date

The definition of asthma employed in our study was either: (1) a hospital discharge for asthma (ICD9 493); or (2) two medical fee-for-service claims coded ICD9 493 within a moving 12-month period; or (3) two prescriptions for a known asthma medication within a moving 12-month period. The *index date *was defined as the date of the first episode by which the diagnosis of asthma was established. Previous research comparing various database asthma definitions to a gold standard of an allergist's diagnosis of childhood asthma found that this definition of asthma had a positive predictive value of 92%, which was higher than other definitions requiring fewer visits and prescriptions [17,18].

### Statistical Analysis

#### Antibiotic rates in asthmatics versus controls

Rates of antibiotic dispensing in asthmatics and non-asthmatics by age 6 were examined in those with at least 6 years of follow-up from birth (n = 128,872). We defined five groups according to their asthma diagnosis in this period: 'no asthma by age 6' or asthma definition reached at age 2, 3, 4, and 5 years. Asthmatics reaching the index date within their first two years of life (n = 22,736) were excluded since the diagnosis of asthma is difficult and confounded by other illnesses and the rate of antibiotic dispensing was high and variable in this age range. Annual antibiotic dispensing rates until age 6 years were calculated in these five cohorts. As previous work has shown changes in antibiotic prescribing rates in this population over time [12], we adjusted for calendar year in all analyses. Rate ratios for antibiotic dispensing in the four asthma groups compared to the 'no asthma by age 6' reference group were also plotted.

#### Antibiotic rates near the asthma index date

We examined incident asthma cases identified prospectively from age two years until the earliest of: 31st December 2005, death, or departure from BC (n = 18,864). For children not registered in the MSP on 31st December 2005, the date of departure from BC was defined as the latest occurrence of: MSP coverage, MSP visits, hospital separations, or PharmaNet records. This allowed follow-up up to capture asthma diagnosed to a maximum age of 8 years. The monthly rates of antibiotic and asthma medication dispensing were plotted by age at asthma index date in those followed for 6 months prior to and also for 6 months post the asthma index date (n = 17,297).

Poisson regression analysis was used to estimate rate ratios (RR) and their 95% confidence intervals (CI) for the rate of antibiotic dispensing in the six months prior to the index date adjusting for the various potential confounders. As the number of antibiotics dispensed per child was overdispersed, the deviance chi-square was used to adjust the standard errors. Potential confounders adjusted for included gender, birth weight, and congenital anomalies. Time varying covariates included calendar year, seasonality, child age, and in the preceding year: quintiles of socioeconomic status (SES), frequency of physician visits, and allergist/respirologist/immunologist visit.

To calculate the RR and 95% CI for the antibiotic prescribing rate in the month before the index date compared to the 5 months before that, Poisson models were used with general estimating equations to obtain robust standard errors accounting for the correlation between the two rates being estimated for each child [19]. Interactions between the RR and all of the confounders were tested in a Poisson model and results were presented for confounders that independently significantly altered this RR.

To examine indications for antibiotics prescribing in the six months prior to the index date, the infectious diagnosis (according to ICD9 codes) were reported for the most temporal MSP billing code within 5 days preceding the dispensing episode. Proportions of the indications for antibiotic prescribing in the month prior to the index date and the 5 months prior to that were compared via chi-square tests.

## Results

### Antibiotic rates in asthmatics versus controls

For those 106,136 children with at least 6 years of follow-up (excluding asthmatics reaching their index date in the first 2 years of life), 96,496 (91%) did not meet the definition of asthma by age 6 years and the remaining met the definition at age 2 (2.6%), 3 (2.4%), 4 (2.1%) and 5 (1.9%). The asthmatics first met the criteria for diagnosis in 57% of cases via a medical claim, 53% by prescription, and 5% by hospital discharge. 12% met the criteria for asthma diagnosis by medical claim and prescription simultaneously. The rates of antibiotic prescribing by year of age and by age at the asthma index date (adjusted for calendar year - presented for year 2000) were characterized for these children (Table 1). The rate was elevated in the asthmatics compared to the non-asthmatics and this ratio peaked during the year of asthma diagnosis with a RR of around 2.30-2.36 (Table 1).

**Table 1:** Rates of antibiotics dispensed per year of age, by age of asthma diagnosis and rate ratios compared to non-asthmatics by age 6 (rates standardized for year 2000)

	No asthma by age 6 (n = 96,496)	Age at asthma index date							
		
		Age 2 (n = 2,791)		Age 3 (n = 2,572)		Age 4 (n = 2,238)		Age 5 (n = 2,039)	
	
Age, years	Rate of ABX per 100 PY	Rate of ABX per 100 PY	RR (95% CI)*	Rate of ABX per 100 PY	RR (95% CI)*	Rate of ABX per 100 PY	RR (95% CI)*	Rate of ABX per 100 PY	RR (95% CI)*
0	84.6	123.9	1.47 (1.40-1.53)	118.5	1.40 (1.34-1.47)	110.8	1.31 (1.25-1.38)	108.3	1.28 (1.22-1.35)
1	118.8	190.1	1.60 (1.54-1.66)	173.4	1.46 (1.40-1.52)	165.0	1.39 (1.33-1.45)	156.4	1.32 (1.26-1.46)
2	90.1	213.0	2.36 (2.28-2.45)	149.0	1.65 (1.58-1.73)	140.2	1.56 (1.48-1.63)	124.9	1.39 (1.32-1.46)
3	89.0	170.0	1.91 (1.84-1.99)	207.5	2.33 (2.24-2.42)	156.8	1.76 (1.68-1.84)	136.9	1.54 (1.46-1.62)
4	85.7	148.1	1.73 (1.65-1.80)	162.1	1.89 (1.81-1.97)	197.0	2.30 (2.20-2.40)	141.9	1.66 (1.57-1.74)
5	85.8	143.3	1.67 (1.60-1.75)	147.2	1.72 (1.64-1.79)	164.0	1.91 (1.83-2.00)	198.1	2.31 (2.21-2.41)
									
Before age 6	91.5	162.5	1.77 (1.75-1.80)	156.7	1.72 (1.69-1.74)	152.2	1.66 (1.63-1.69)	141.0	1.55 (1.51-1.58)

* compared to rate of ABX in those without asthma by age 6 and adjusted for calendar year

Abbreviations: ABX - antibiotics; PY - person years; RR - rate ratio

### Antibiotic rates near the asthma index date

**Figure 1:**
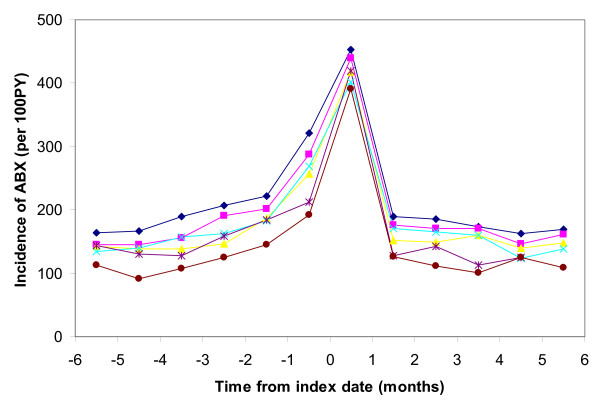
Monthly rate of antibiotic medication dispensing per 100 person-years by months before and months after asthma index date and by age at asthma index date* (Age (years) at asthma index date: blue diamonds = 3, pink squares = 4, yellow triangles = 5, turquoise crosses = 6, purple stars = 7, brown circles = 8-9). * N = 17,297 with 6 months follow-up before and 6-months follow-up post asthma index date

**Figure 2:**
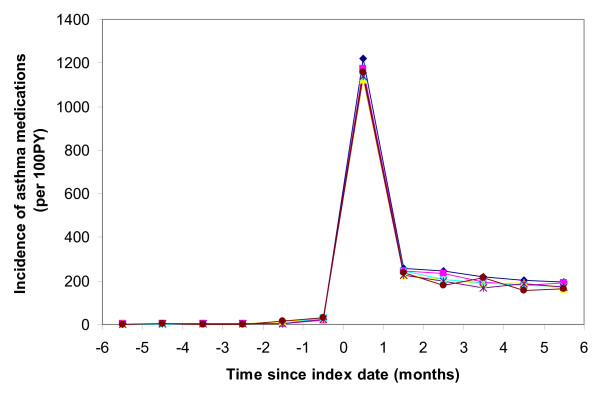
Monthly rate of asthma medication dispensing per 100 person-years by months before and months after asthma index date and by age at asthma index date* (Age (years) at asthma index date: blue diamonds = 3, pink squares = 4, yellow triangles = 5, turquoise crosses = 6, purple stars = 7, brown circles = 8-9). * N = 17,297 with 6 months follow-up before and 6-months follow-up post asthma index date

A total of 18,864 children first met our definition of asthma between their 2nd birthday and 31 December 2005. Antibiotics were dispensed to 9,841 (52%) of these children in the 6 months leading up to their asthma index date. They were dispensed 17,245 courses of antibiotics during this period and 4,293 (25%) of these antibiotics were dispensed in the month before the index date. The three most frequently antibiotics/type of antibiotics dispensed were amoxicillin (45%), macrolides (29%) and cephalosporins (14%). Antibiotic dispensing increased prior to the asthma index date and rapidly decreased in the 6 months afterwards (Figure 1). Very few asthma medications were dispensed prior to the asthma index date, a large rate dispensed on the asthma index date (61% of asthmatics dispensed asthma medications on the index date), and then a level similar to that of antibiotic dispensing afterwards (around 200 per 100 PY) (Figure 2).

Exposure to antibiotics in the first year of life was associated with increased antibiotic dispensing in the 6 months prior to the index date (RR 1.26, 95% CI 1.22-1.30) in the multivariable model (Table 2). In addition, visiting a specialist in the previous year (RR 0.85, 95% CI 0.75-0.96) and congenital anomalies (RR 0.93, 95% CI 0.88-0.98) were associated with lower antibiotic dispensing. Asthma diagnosed in spring and summer was associated with a greater rate of antibiotics being dispensed in the previous 6 months than asthma diagnosed in the winter or fall. A decreasing rate of antibiotics dispensing was observed across increasing SES quintiles with the highest SES quintile being associated with 0.91 (95% CI, 0.86-0.96) fold fewer antibiotics being dispensed. Also an increasing rate of dispensing was observed across increasing frequencies of physician visits in the last year, so those visiting a physician ≥ 12 times in the previous year were dispensed antibiotics 7.79 (95% CI, 7.13-8.51) times more frequently relative to those visiting a physician <12 times.

**Table 2:** Multivariable rate ratios of antibiotic dispensing in six months prior to the index date

Confounder	n	%	Rate of ABX per 100 PY (6 months prior to diagnosis)	RR	95% CI	p
Age at asthma diagnosis						
2	6,254	33.2	209.9	1.00		<.0001
3	4,810	25.5	185.5	1.03	0.98 - 1.07	
4	3,316	17.6	164.4	1.00	0.95 - 1.05	
5	2,338	12.4	171.9	1.10	1.04 - 1.17	
6	1,272	6.7	153.9	1.03	0.96 - 1.12	
7-8	874	4.6	117.4	0.89	0.81 - 0.99	
Year of asthma diagnosis						
1999	475	2.5	226.9	1.00		<.0001
2000	1,484	7.9	207.8	0.98	0.88 - 1.09	
2001	2,165	11.5	206.0	1.03	0.93 - 1.14	
2002	2,924	15.5	178.0	0.95	0.86 - 1.06	
2003	3,494	18.5	180.4	0.97	0.88 - 1.07	
2004	4,351	23.1	174.2	0.98	0.89 - 1.08	
2005	3,971	21.1	171.3	1.05	0.95 - 1.16	
Season of asthma diagnosis						
Winter	5,085	27.0	179.9	1.00		<.0001
Spring	5,140	27.2	217.8	1.24	1.19 - 1.30	
Summer	3,142	16.7	194.9	1.13	1.07 - 1.18	
Fall	5,156	27.3	150.6	0.85	0.81 - 0.89	
Antibiotic exposure in first year						
No	8,542	45.3	145.6	1.00		<.0001
Yes	10,322	54.7	213.8	1.26	1.22 - 1.30	
Gender						
Female	7,762	41.2	181.0	1.00		0.38
Male	11,102	58.9	184.3	0.99	0.95 - 1.02	
Allergist/respirologist/immunologist visit (in last year)						
No	18,487	98.0	182.7	1.00		0.008
Yes	377	2.0	196.7	0.85	0.75 - 0.96	
Congenital anomalies (in first year)						
No	16,943	89.8	182.0	1.00		0.006
Yes	1,921	10.2	190.9	0.93	0.88 - 0.98	
SES in the year before diagnosis (quintiles of whole population)*						
First quintile	4,357	23.1	201.6	1.00		0.004
Second quintile	4,074	21.6	189.8	0.98	0.94 - 1.03	
Third quintile	3,744	19.9	185.4	0.98	0.93 - 1.03	
Fourth quintile	3,290	17.4	170.0	0.95	0.90 - 1.00	
Fifth quintile	2,744	14.6	157.9	0.91	0.86 - 0.96	
Unknown	655	3.5	171.3	1.05	0.96 - 1.15	
Physician visits in last year						
0-2	2,838	15.0	44.3	1.00		<.0001
3-4	3,147	16.7	92.5	2.07	1.87 - 2.29	
5-9	4,626	24.5	147.3	3.31	3.02 - 3.62	
10-12	4,308	22.8	220.7	4.91	4.49 - 5.37	
>12	3,945	20.9	355.3	7.79	7.13 - 8.51	
Birth weight (kg)						0.92
< 1.5	177	0.9	185.3	1.00		
1.5 - 2.5	923	4.9	195.2	1.01	0.85 - 1.21	
2.5 - 3.5	9,593	50.9	185.6	1.01	0.86 - 1.19	
3.5 - 4.5	7,748	41.1	179.0	1.03	0.87 - 1.22	
> 4.5	401	2.1	169.6	1.01	0.83 - 1.23	
Unknown	22	0.1	127.3	0.93	0.52 - 1.66	

Abbreviations: ABX - antibiotics; PY - person years; RR - rate ratio

* Socioeconomic status by neighbourhood income quintiles - the fifth is the highest income quintile and the first is the lowest income quintile.

The rate of antibiotic dispensing one month prior to the index date was elevated (RR 1.66, 95% CI 1.60-1.71) compared to the immediate 5 months before this (Table 3). Gender, season, SES, visit to a specialist, and frequency of physician visits in the year prior to diagnosis all independently modified this RR. Specifically, in the subgroup analysis, the rate of antibiotic dispensing in girls and boys was significantly higher in the one month prior to the index date as compared to the 5 months prior (RR 1.77, 95% 1.68-1.87 and 1.58; 95% CI 1.51 - 1.65, respectively). Similarly, those who had not visited a specialist in the year prior had a higher ratio (RR 1.67, 95% 1.61-1.73) relative to those that had. The RR was greater in the fall and winter than spring and summer, although all seasons were associated with a significantly greater rate of antibiotic dispensing one month prior to the index rate than in the previous 5 months. The ratios were also higher for those with fewer physician visits in the prior year and those with a lower SES. For example, those with ≤2 physician visits (RR 2.22, 95% CI 1.86-2.64) and those in the lowest SES quintile (RR 1.70, 95% CI 1.59-1.82) had a higher rate of antibiotic dispensing in the one month prior to the index date compared to the 5 months prior.

**Table 3:** Rate ratios of the frequency of antibiotic dispensing in month before the index date compared to 5 months before in significant subgroups

			Rate of ABX per	Rate of ABX per	Rate of ABX in month before diagnosis vs 5 months before		
			100 PY (month	100 PY (1-6 months			
Subgroup	n	%	before diagnosis)	before diagnosis)	RR	95% CI	p
Overall	18,864	100.0	273.1	164.9	1.66	1.60 - 1.71	<.0001
Gender							
Female	7,762	41.2	284.5	160.4	1.77	1.68 - 1.87	<.0001
Male	11,102	58.9	265.1	168.1	1.58	1.51 - 1.65	<.0001
Season of asthma diagnosis							
Winter	5,085	27.0	336.3	155.1	2.17	2.04 - 2.30	<.0001
Spring	5,140	27.2	290.9	202.9	1.43	1.35 - 1.53	<.0001
Summer	3,142	16.7	210.8	191.7	1.10	1.00 - 1.21	0.04
Fall	5,156	27.3	249.0	131.2	1.90	1.77 - 2.04	<.0001
Allergist/respirologist/immunologist visit (in last year)							
No	18,487	98.0	274.1	164.4	1.67	1.61 - 1.73	<.0001
Yes	377	2.0	213.2	193.4	1.10	0.80 - 1.51	0.54
Physician visits in last year (quintiles)							
0-2	2,838	15.0	81.6	36.8	2.22	1.86 - 2.64	<.0001
3-4	3,147	16.7	155.2	80.0	1.94	1.73 - 2.18	<.0001
5-9	4,626	24.5	228.3	131.2	1.74	1.61 - 1.88	<.0001
10-12	4,308	22.8	336.2	197.6	1.70	1.59 - 1.82	<.0001
>12	3,945	20.9	488.5	328.6	1.49	1.41 - 1.57	<.0001
SES in the year before diagnosis (quintiles of whole population)							
First quintile	4,357	23.1	204.5	120.4	1.70	1.59 - 1.70	<.0001
Second quintile	4,074	21.6	185.6	114.8	1.62	1.51 - 1.62	<.0001
Third quintile	3,744	19.9	195.9	109.1	1.80	1.66 - 1.80	<.0001
Fourth quintile	3,290	17.4	165.1	103.0	1.60	1.47 - 1.60	<.0001
Fifth quintile	2,744	14.6	146.4	97.1	1.51	1.36 - 1.51	<.0001

Abbreviations: ABX - antibiotics; PY - person years; RR - rate ratio

* Socioeconomic status by neighborhood income quintiles - the fifth is the highest income quintile and the first is the lowest income quintile.

Upper and lower respiratory tract infections (RTIs), otitis media and bronchitis were the most frequent diagnoses preceding the antibiotic dispensations in the 6 months prior to the index date (Table 4). Lower RTI and bronchitis were more frequently associated with antibiotic dispensing in the one month before the index date compared to the 5 months before. Upper RTI, acute otitis media, lower urinary tract infection, and skin/soft tissue infection were less frequently associated with antibiotic dispensing in the one month before the index date compared to the 5 months before that.

**Table 4:** Most common diagnoses related to antibiotics dispensed in the 6 months prior to the index date (N = 17,245 antibiotic prescriptions)

	One month before		1-6 months before		Six months before	
	
Diagnosis (ICD9 code)	Number of antibiotic prescriptions	%	Number of antibiotic prescriptions	%	Number of antibiotic prescriptions	%
Upper respiratory tract infection (034, 461-465)	993	23.1	3062	23.6	4055	23.1
Bronchitis (466, 490-491)*	785	18.3	1658	12.8	2443	13.9
Acute otitis media (381-382)*	544	12.7	2140	16.5	2684	15.3
Lower respiratory tract infection (481-486)*	211	4.9	386	3.0	597	3.4
Skin/soft tissue infection (035, 680-686)*	39	0.9	205	1.6	244	1.4
Lower urinary tract infection (595, 597, 599)*	25	0.6	206	1.6	231	1.3
Asthma (493)	9	0.2	20	0.2	29	0.2

* p < 0.01 for chi-square test for difference in proportion of antibiotics dispensed due to that diagnosis in the previous month compared to 5 months before that.

## Discussion

This is the first study to clearly show a pattern of increased consumption of antibiotics in children in the period immediately before the diagnosis of asthma. Those children who were eventually diagnosed with asthma had a higher rate of antibiotic use than those who were never diagnosed with asthma during the follow up period of the study. However, the rate of antibiotic use was at the highest in the year that asthma was diagnosed. Specifically, in the one month period leading up to their first asthma-related episode, children with asthma were almost 1.7 times more likely to receive an antibiotic prescription than in the five months prior. In addition, these children were more likely to receive a diagnosis of a lower respiratory tract infection or bronchitis rather than asthma in this time period. This finding, coupled with the fact that those children that were ultimately diagnosed with asthma had a higher baseline rate of antibiotic consumption, highlights the need for clinicians to diagnosis asthma in children promptly and not to inappropriately prescribe antibiotics, especially in those with respiratory symptoms.

Antibiotics are an important component of treating bacterial infections in both adults and children. But their overuse has important public health consequences as it leads to drug resistance [20-24]. Several studies have showed that antibiotic use in Canadian children is not only higher than their European counterparts but that penicillins are more likely to be prescribed in Europe compared to macrolides in Canadian children [11,12]. Furthermore, clinicians should also keep in mind that young children who present with symptoms of shortness of breath and wheezing may have an infection but for the most part it is viral rather than bacterial, negating the need for antibiotics [25]. As such, much of the antibiotic use documented in this study may have been avoidable. Given the high probability of cough and shortness of breath in young children being due to a viral infection or possibly non-infectious cause (e.g., asthma), a "watch and wait" approach [26-28] is advocated for upper respiratory tract infections and frequently for otitis media. Asthma guidelines have been shown to be effective in controlling antibiotic prescribing in this regard but are often challenging to implement [29].

It is well known that the diagnosis of asthma in children less than 5 years of age is difficult and related to the fact that episodic wheezing and shortness of breath are also present in children who do not have asthma [30]. In addition, there is a challenge to objectively measuring airflow obstruction in this age group especially in primary care where access to spirometry in all age groups is not readily available. Thus, guidelines suggest the following as having a high positive predictive value of asthma in children - frequent (more then once a month) episodes of wheeze, activity induced cough or wheeze, nocturnal cough when the child does not have a viral infection, absence of seasonal variation in wheeze and persistence of respiratory symptoms after the age of 3 years. In young children, the diagnosis of asthma is based on a clinician's judgment, symptomatic improvement after a trial of therapy with inhaled corticosteroids and use of a short acting beta-agonist for symptom relief. In our study, we partially circumvented the problem by excluding all asthma diagnoses in the first two years of life. In addition, the patterns of antibiotic use were the same for older children (5 years old and greater) as younger children (< 5 yrs) increasing the confidence of our results in younger children.

Several of our observations require further thought. The strongest associations occurred in children from lower socioeconomic status. Our data do not allow for a definitive statement regarding this phenomenon. However, since higher socioeconomic status is associated with higher education, it is possible that parents of higher socioeconomic status were more inquisitive of the child's care leading to the consideration of alternate diagnoses other than infection. Also, we found that females were more likely to have a higher number of prescriptions for antibiotics prior to the diagnosis of asthma. Given that asthma is more common in prepubescent boys than in girls, this result seems counterintuitive. However, given this difference in incidence physicians may be even more reluctant to consider asthma as a diagnosis for girls in this age group and therefore are more likely to prescribe more antibiotics. Another finding of note included that exposure to antibiotics in the first year of life was associated with increased antibiotic dispensing in the 6 months prior to the index date. Despite the fact that the frequency of physician visits were adjusted for in the analysis, it is possible that this finding might be due to residual confounding due to treatment seeking behavior. Finally, we found that asthma diagnosed in spring and summer was associated with a greater rate of antibiotics being dispensed in the previous 6 months than asthma diagnosed in the winter or fall. A possible explanation for this finding is that, due to the presence of allergens in the spring and summer, clinicians might experience patients with more severe respiratory infections and can easily rule out seasonal influenza as the cause. As such, more antibiotics are prescribed during these seasons. All of these findings merit further examination in well-conducted prospective studies.

Our study is not without limitations. Due to the nature of the data, many important potential confounders (such as birth order, family history of allergic disease, maternal smoking) could not be adjusted for in the multivariable models. However, these variables are unlikely to impact the main analysis which showed an increased rate of antibiotic prescription just before the diagnosis of asthma.

There are many ways to define asthma diagnosis in administrative data [31-33]. We employed a method that was developed and validated by researchers in Canada although on a slightly older group of asthmatics [18]. However, it is possible that, by using this method, we have misclassified some children as asthmatics whose symptoms subsequently resolve. In addition, the converse is also possible with some asthmatic children being classified as non-asthmatic. It's likely there is physician and possibly parental reluctance, to consider asthma as a diagnosis given the potential implications such a label brings. For example, Their et al showed that physicians were very likely to prescribe inhaled corticosteroids for children with asthma but only a small minority of patients had their prescription filled [34]. As such, these patients might be misclassified in an administrative database study.

We acknowledge that the diagnosis of asthma is difficult in those less than 5 years of age but believe that the consistency of our results between the younger (< 5years) and older (> or = to 5 years) is a validation of our definition. Although we document an association between antibiotic use and the diagnosis of asthma, our study does not address the issue of whether asthma is caused by antibiotics or is an associated finding. Clarifying the answer to this question would be difficult to prove conclusively without a clinical trial which would obviously be prohibited by ethical issues. However, the pattern of use of antibiotics and the immediate reduction in their use post-asthma diagnosis suggests that antibiotics were being used for asthmatic wheeze. This assumption is strengthened by considered the reasons for antibiotic prescription leading up to the asthma diagnosis (bronchitis and upper respiratory infections).

## Conclusions

Our study shows an increased use of antibiotics in children ultimately diagnosed with asthma compared to children without this diagnosis and use of antibiotics is highest just before the diagnosis of asthma. This suggests that clinicians are initially slow to respond to a pattern of cough as being due to asthma but rather label it as an acute infection. In the setting where antibiotics are often prescribed, a wait and see approach and earlier consideration of the possibility of asthma would lead to a faster resolution of symptoms and the establishment of a correct diagnosis. Less use of antibiotics would also facilitate a lower risk for the development of bacterial resistance as well as the avoidance of antibiotic related morbidity linked to adverse events. We therefore propose that the prescription of more than two courses of antibiotics for respiratory symptoms in a child over a six month period should be sufficient to warrant consideration of an asthma diagnosis.

## Competing interests

The author declares that they have no competing interests.

## Authors' contributions

FM was involved in the design of the study, submission of ethics, interpretation of the data, and created the first draft of the manuscript. CM designed the study, interpreted the data and created the first draft of the manuscript. KR, is a statistician who was analyzed the data and helped with data interpretation. LL and MF conceived the study and were involved in data interpretation. All authors read and approved the final manuscript.

## Funding

Funding for this study was provided by the BC Lung Association, British Columbia, Canada. Dr. Carlo Marra is a Canada Research Chair in Pharmaceutical Outcomes and a Michael Smith for Health Research (MSFHR) Scholar. Dr. Larry Lynd is a CIHR New Investigator and a MSFHR Research Scholar. Dr. Mark FitzGerald is a MSFHR Distinguished Scholar and a CIHR/BC Lung Association Investigator Award.

## Pre-publication history

The pre-publication history for this paper can be accessed here:

http://www.biomedcentral.com/1471-2466/11/32/prepub
